# Predicting Pain: Electroencephalography Signatures of Neural Integration During Experimental Tonic Thermal Pain

**DOI:** 10.1002/ejp.70313

**Published:** 2026-06-30

**Authors:** Yiyuan Han, Elia Valentini, Sebastian Halder

**Affiliations:** ^1^ School of Computer Science and Electronic Engineering University of Essex Colchester UK; ^2^ Department of Neurological Surgery and UCSF Weill Institute for Neurosciences University of California San Francisco California USA; ^3^ Department of Psychology and Centre for Brain Science University of Essex Colchester UK

## Abstract

**Background:**

The identification of reliable neural signatures for pain remains a critical challenge in both clinical and experimental settings. While electroencephalography (EEG) provides a promising avenue for pain assessment, it remains unclear whether phase‐ or power‐based neural integration drives pain‐state discrimination. This study investigates functional connectivity and cross‐frequency coupling (CFC) as candidate signatures of neural integration for pain prediction.

**Methods:**

We recorded 62‐channel EEG data from 36 healthy participants across experimental conditions, including tonic thermal pain, non‐painful warm stimulation and resting states. Functional connectivity within the alpha band and CFC across delta, theta, alpha and low‐beta bands was computed using phase‐ and power‐based measures. Machine learning models were trained to classify pain from non‐painful conditions, with prediction accuracy serving as an index of neural integration performance.

**Results:**

Phase‐based features outperformed power‐based features in tonic thermal pain prediction, which indicated a dominant role for phase synchrony. The strongest topographical signatures are the connectivity involving frontal and occipital regions driven by enhanced alpha‐phase connectivity or theta‐alpha/delta‐theta cross‐frequency coupling.

**Conclusions:**

Our findings demonstrate that phase‐based neural integration outperforms power‐based integration in characterising tonic pain, and that the inclusion of amplitude information actively reduces discriminability. Phase‐based measures of functional connectivity in the alpha band and cross‐frequency coupling with other low‐frequency oscillations may serve as candidate EEG signatures of pain, offering a data‐driven framework with potential applications in research and clinical contexts.

**Significance Statement:**

This study introduces an EEG‐based framework for tonic pain prediction that integrates multiple neural signatures of integration. High classification accuracy between painful and non‐painful states provides new machine learning–driven insight into phase‐based neural integration, reflected in functional connectivity and CFC. The findings broaden theoretical perspectives on pain processing within machine learning approaches and underscore clinically relevant potential for developing reliable, noninvasive tools to improve pain assessment.

## 1 Introduction

Pain imposes major health and societal burdens worldwide (Macchia et al. 2025). Clinically, pain is usually inferred from verbal or behavioural reports (Beecher 1959; Noble et al. 2005), which are unavailable in patients who cannot communicate (e.g., disorders of consciousness, anaesthesia and infants). In such contexts, electroencephalography (EEG) offers a promising tool to identify neural signatures apt to provide insight into a state of pain (Chatelle et al. 2014), combining temporal resolution with non‐invasive bedside applicability (Levitt and Saab 2019; Nir et al. 2010; van der Miesen et al. 2019).

The experience of pain reflects the integration of cognitive, emotional and motivational processes across distributed brain networks (Apkarian et al. 2011; Merskey and Bogduk 1994; Ploner et al. 2017). Studying neural integration may thus reveal pain signatures of the subjective experience. Oscillatory activity is a particularly suitable candidate, as it enables quantification of inter‐regional or intra‐regional communication. Functional connectivity captures statistical dependencies between regions (Stam et al. 2006), while cross‐frequency coupling (CFC) indexes integration across rhythms (Liu et al. 2015).

Neuronal oscillations, which give rise to both functional connectivity and CFC, underpin neural integration and are linked to pain across frequency bands (Nickel et al. 2017; Ploner et al. 2017). Gamma event‐related synchronisation (ERS) following nociceptive stimuli has been proposed as pain‐specific (Zhang et al. 2012), but concerns remain about artefacts (Muthukumaraswamy 2013; Valentini et al. 2021). By contrast, alpha activity is less affected by artefacts. The relationship between resting peak alpha frequency and pain sensitivity is not uniform across studies: while Furman et al. (2018) reported that slower peak alpha frequency is linked to high pain sensitivity, others reported the opposite direction (De Martino et al. 2021; Nir et al. 2010), while Valentini et al. (2022) suggested that individual alpha frequency does not play a causal role in pain experience. Yet both gamma ERS and peak alpha frequency are spatially localised (Chowdhury et al. 2025; Furman et al. 2018; Nir et al. 2010). The spectral profile of chronic pain differs from experimental pain (Mussigmann et al. 2022). Hence, we focus the present study on the tonic experimental context.

EEG‐based pain prediction has taken two broad approaches to feature extraction. The first uses raw spectral or spatial features, such as power or amplitudes at individual electrodes input into classifiers (Wang et al. 2020; Yu et al. 2020). The second derives relational metrics that capture how brain regions or neuronal oscillations interact, such as connectivity or coupling indices (Hsiao et al. 2021; Levitt et al. 2020). The first approach is computationally simple but treats each channel independently, which misses the coordinated, network‐level activity characterising pain. Our study takes the second path: we extracted integration features, including alpha‐band inter‐site phase clustering (ISPC) for spatial integration, and phase‐ and power‐based CFC for oscillatory integration. Crucially, whether phase or amplitude integration drives coherence‐based pain associations has not been directly tested. We used machine learning to evaluate candidate neural‐integration signatures, using prediction accuracy as a proxy for informativeness.

## 2 Methods

### 2.1 Participants

This study was approved by the Ethics Committee of the University of Essex. Forty‐three healthy participants entered the study. All participants had normal or corrected‐to‐normal vision and hearing. Before the experiment, all participants completed a screening form and were asked to disclose their history of neurological, psychiatric or pain disorders; this led to the exclusion of two participants. Five other participants were excluded due to procedural failures and technical issues. Data from 36 participants were eventually analysed (19 female, mean: 24.75, range: 20–56). Of these, 24 self‐identified as white/Caucasian, six as Asian/Pacific Islander, and six as Other ethnic group. The details of the seminal study can be found in (Valentini et al. 2022).

### 2.2 Experimental Design

#### 2.2.1 Experimental Conditions

The experimental design included five conditions: two thermal (hot [H] and warm control [W]), two resting states (eyes‐open [O] and eyes‐closed [C]) and one auditory condition (sound [S]). The hot condition (H) induced tonic thermal pain, whereas the warm control condition (W) served as an innocuous sensory control. The eyes‐open condition (O) represented a baseline resting state dominated by visual input, while the eyes‐closed condition (C) represented a baseline resting state with minimal sensory input. Together, these conditions enabled a comprehensive assessment of neural integration underlying sensory and pain processing. The auditory condition (S) was excluded from the present analysis to reduce complexity.

#### 2.2.2 Experimental Procedure

EEG was recorded with 62 Ag/AgCl electrodes (Easycap, BrainProducts GmbH, Gilching, Germany), which were placed according to the 10–20 system. The impedance of all electrodes was kept below 10 kΩ and the EEG signal was digitised at 1000 Hz (Synamps RT, Neuroscan, Compumedics). The reference was on the left earlobe and the ground on the AFz electrode. Offline we re‐referenced the data to the participant's right earlobe.

Each condition lasted 5 min. The hot condition (H) consisted of a prolonged immersion of the participants' left hand, up to the wrist, in a 30‐L tank (RW‐3025P, Medline Scientific) with circulating water at a temperature of $44.5{\left(\pm 0.49\right)}^{{}^{\circ}}C$. During this phase, the initial setting started from $45^{{}^{\circ}}C$, which is known to induce a moderate level of pain in healthy individuals (Granot et al. 2008). The warm control condition (W) had the same settings as the hot condition (H), except that the temperature was $6^{{}^{\circ}}C$ lower.

Participants were seated 65 cm from a screen, with their left hand placed in the water bath and their right hand used to control a mouse and volume adjustment knob. They were asked to focus on the unpleasantness of sensory stimulation and report it on a visual analogue scale (VAS) with anchors 0 (*no unpleasantness*), 25, 50, 75 and 100 (*intolerable unpleasantness*).

If the participant could not tolerate the initial temperature, this was reduced by $0.5^{{}^{\circ}}C$. Conversely, if the VAS was rated below 50, the temperature was increased by $0.5^{{}^{\circ}}C$. After adjusting the temperature, the calibration phase was repeated until the participant consistently rated unpleasantness between 50 and 75. Following this procedure the EEG recording started with two 2.5‐min resting state blocks each (eyes‐open and eyes‐closed), which were also recorded after the sensory conditions (Valentini et al. 2022).

### 2.3 EEG Pre‐Processing

The signal was down‐sampled from 1000 to 500 Hz, the DC components were removed with a high‐pass filter with a cut‐off frequency of 0.5 Hz, and sinusoidal artefacts related to the power line were removed with a 50 Hz band‐stop filter. Eye‐blink and muscle‐movement artefacts were removed using independent component analysis (ICA; Makeig et al. 1996), implemented in EEGLAB via the extended infomax algorithm (runica). ICA components were identified for rejection based on two criteria applied through visual inspection: (1) a scalp topography characteristic of ocular activity, where a frontal distribution had maximal amplitude at Fp1/Fp2 and (2) a time‐series waveform consistent with muscle activity, characterised by broadband high‐frequency bursts. The remaining components, reflecting neural brain activity, were retained and the EEG signal was reconstructed. Full preprocessing details follow the procedure described in the seminal study using these data (Valentini et al. 2022).

**FIGURE 1:**
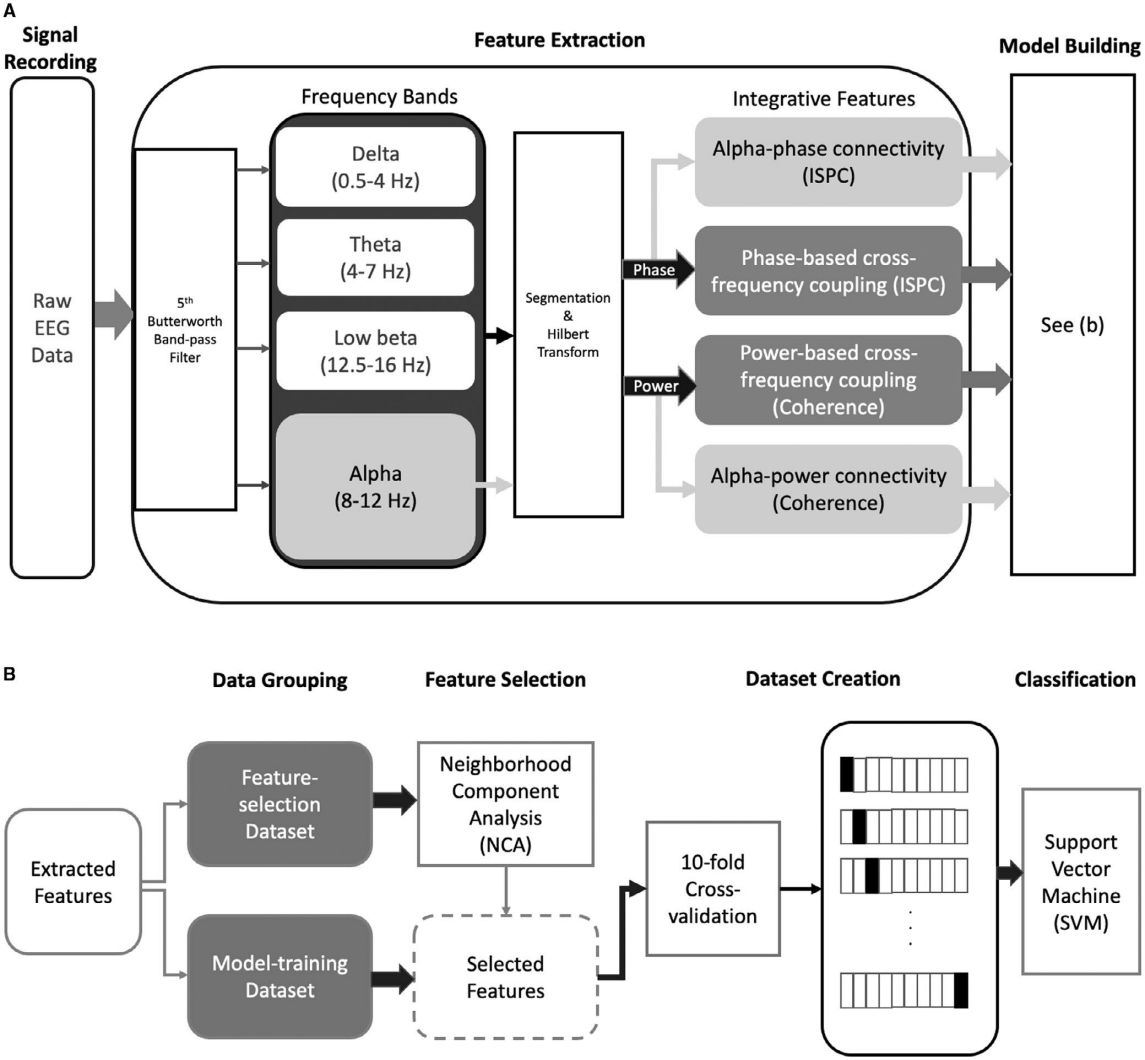
(A) Signal processing pipeline: First, delta (0.5–4 Hz), theta (4–7 Hz), alpha (8–12 Hz) and low beta (12.5–16 Hz) bands were extracted from the EEG data. We then computed power and phase using the Hilbert transform. Then, we extracted neural signatures representing functional connectivity or cross‐frequency coupling. (B) Model training and testing pipeline: We split the data into two groups. While one dataset was used for feature selection, the other was used to train and test the classifiers with the selected features using 10‐fold cross‐validation, where the folds are cut in the original temporal order to avoid data leakage. Subsequently, feature‐selection and model‐training datasets were swapped.

To investigate how trial lengths affected prediction accuracy, we segmented the data into 1, 2.5, 5 and 10 s(s) trials (Fraschini et al. 2016), where the short trials increase the number of trials available, which can benefit machine learning. While the longer trials can make the measures more stable, since each segment carries more oscillations at each band. To make the dataset as large as possible, we set the overlap ratio between two neighbouring trials to 50%. Therefore, the number of trials in each condition from each participant was approximately 300 (1 s), 240 (2.5 s), 120 (5 s), and 60 (10s). EEG signals were transformed using current source density (CSD) to reduce signal distortion caused by volume conduction and produce a sharper and more distinct topography, Appendix A1.1 describes the mathematical principle of CSD (Kayser and Tenke 2015; Mitzdorf 1985). Before feature extraction, the signals were band‐pass filtered by a fifth‐order Butterworth filter (zero‐phase, no phase distortion) into four frequency bands: delta (0.5–4 Hz), theta (4–7 Hz), alpha (8–12 Hz) and low beta (12.5–16 Hz); the order of 5 was selected according to the fact that Butterworth filter is an Infinite Impulse Response (IIR) filter. Then we applied the Hilbert transform to calculate the power and phase of the signals (see Figure 1a).

### 2.4 Functional Connectivity and CFC

Because many metrics exist (Bott et al. 2023), we compared integration features based on our phase versus power focus. Synchrony can be defined as phase–phase, power–power or phase–power interactions (Nickel et al. 2020; Stam et al. 2007). We restricted analysis to phase–phase and power–power measures to isolate their contributions.

Neural signatures of integration were then generated from the calculation of phase (ISPC) or power series (spectral coherence). We investigated functional connectivity only in the alpha band and CFC between two frequency bands from each electrode as mentioned in Section 1. Therefore, we simplified the comparisons to functional connectivity versus CFC, and power versus phase. Specifically, we measured functional connectivity features using the difference of phase or power between two channels, that is, alpha‐phase connectivity (PhaCon) and alpha‐power connectivity (PowCon). In terms of CFC, we measured various pairs of frequency coupling (across delta, theta, alpha and low‐beta bands) per each EEG channel and for both phase and power, that is, phase‐based CFC (PhaCou) and power‐based CFC (PowCou). To assess the robustness of phase‐based connectivity findings against volume conduction artefacts, the debiased weighted Phase‐Lag Index (dwPLI) was additionally computed for all electrode pairs and frequency band combinations; results are reported in Appendix A4 (Vinck et al. 2011).

#### 2.4.1 Inter‐Site Phase Clustering

ISPC describes the distribution of phase differences between two temporal series from different sources, that is, EEG channels or frequency bands in this study. It is calculated using the following formula:

(1) $$\begin{array}{r} {\mathrm{ISPC}}_{\mathrm{xy}}=\left|\frac{1}{n}\sum_{t=1}^{n}e^{i\left[\phi_{x}\left(t\right)-\phi_{y}\left(t\right)\right]}\right| \end{array}$$

where n represents the total number of time points in each data series, $\phi_{x}$ and $\phi_{y}$ represent the phase angles of two series (i.e., series from two channels for functional connectivity or two frequency bands for CFC). In short, ISPC is the mean exponential value of the imaginary phase angle differences between the signals from two sources in a particular time window, whose values range from 0 to 1.

#### 2.4.2 Magnitude‐Squared Coherence

Coherence is computed via cross‐power spectral density (PSD) between two EEG series from different sources, which reflects the correlation between two series' power distributions:

(2) $$\begin{array}{c} \begin{array}{r} K_{\mathrm{xy}}=\frac{S_{\mathrm{xy}}}{S_{\mathrm{xx}}S_{\mathrm{yy}}} \end{array} \end{array}$$

where $S_{\mathrm{xy}}$ represents the cross PSD between data series *x* and series *y*, and $S_{\mathrm{xx}}$ is the PSD of the signals of series *x*. With the PSD ratio, the coherence between the signals *x* and *y* can be calculated as:

(3) $$\begin{array}{c} \begin{array}{r} {\mathrm{COH}}_{\mathrm{xy}}={\left|K_{\mathrm{xy}}\right|}^{2} \end{array} \end{array}$$

Alternatively, it can be rewritten in a Euler‐like format as the weighted ISPC with the analytic magnitudes (e.g., $m_{x}\left(t\right)$ from channel *x*) as the weights (Cohen 2014):

(4) $${\mathrm{COH}}_{\mathrm{xy}}=|\frac{1}{n}\sum_{t=1}^{n}|m_{x}(t)||m_{y}(t)|e^{i[\phi_{x}(t)-\phi_{y}(t)]}|$$

#### 2.4.3 Topographical Analysis

We calculated the *z*‐scores of the amplitude (i.e., $|m_{x}\left(t\right)|$) and exponential phase (i.e., $e^{i\phi_{x}\left(t\right)}$) for the different conditions. Then we visualised their scalp topography distribution. It is noteworthy that, while the topographical representation of power is straightforward, this is not the case for the visualisation of phase. Because the phase is not a strength measurement, a region's mean or median intensity cannot be calculated arithmetically. However, according to the ISPC formula (1), this issue can be resolved using the mean Euler‐like format of phases in trials calculated as $P_{x}=|\frac{1}{n}\sum_{t=1}^{n}e^{i\left[\phi_{x}\left(t\right)\right]}|$ of the channel *x*, which is defined as the *exponential phase* in this study. To analyse the variation of power/phase distribution, we calculated the topography within each frequency band and condition.

Kullback–Leibler divergence (KLD) quantifies the difference between two probability distributions, for which we analysed the distribution of amplitude or exponential phase values across conditions at each EEG channel. For each participant, KLD was computed per channel between each pair of conditions being compared, then averaged across channels and participants. KLD values were rescaled between 0 and 1 to enable comparison between phase and amplitude measures (see Appendix A1.1, equations 6–7). Higher rescaled KLD indicates greater distributional difference between conditions.

### 2.5 Machine Learning Approach

In this research, all the machine learning evaluations were performed within participants. We trained binary classifiers between all pairwise conditions (with H, W, O and C). We split the pre‐processed data into two sets of the same size in the original order, including one for feature selection and another one of the same size for evaluation. All data trials in both sets were randomly shuffled before further feature selection, training and testing. After feature selection, the excluded set went through training and testing of binary classifiers with 10‐fold cross‐validation. Then the two sets were swapped, and the same process was executed again. We ran all tests on MATLAB (The MathWorks Inc. 2019).

#### 2.5.1 Feature Selection Based on Neighbourhood Component Analysis (NCA)

When considering the number of channels (62) and frequency bands (four) per trial, we could theoretically extract $\sum_{i=1}^{N_{\mathrm{ch}}-1}i=1891$ functional connectivity and $\left(\sum_{i=1}^{N_{f}-1}i\right)N_{\mathrm{ch}}=372$ CFC features, where $N_{\mathrm{ch}}=62$ and $N_{f}=4$ are the number of EEG channels and frequency bands. Clearly, this feature extraction scenario would require significant time expenditure, and numerous features will also increase the necessary number of trials for model training.

To select the most informative features, we used NCA, a supervised method for offline feature selection that assigns a discriminative weight to each feature by optimising a leave‐one‐out nearest‐neighbour classification objective (See Appendix A1.3, Equations A4 and A5) (Yang et al. 2012). The regularisation parameter λ was tuned through cross‐validation. The output is a weight vector w representing the discriminative importance of each feature. We selected the 100 features with the highest weights within each binary classification, based on the preliminary analysis in the Appendix A2.

To determine the features contributing most to each pairwise classification, six binary classifiers were trained across four conditions. Features shared by at least two binary classifiers with one common condition were defined as the representative features of that condition; features shared by all three classifiers involving a condition were interpreted as the most intrinsic neural integration signatures for that condition. For example, features selected via NCA when classifying O versus H, C versus H and H versus W were defined as the representative features of class H.

To prevent information leakage arising from the 50% epoch overlap, the dataset was divided into two halves strictly in temporal order: one half was used exclusively for NCA feature selection, and the other for SVM training and evaluation. Because the split followed the original recording timeline, no overlapping epoch could appear in both halves simultaneously, ensuring that the feature‐selection step had no access to the evaluation data. The two halves were then swapped and the procedure repeated, providing two independent evaluations. To determine the features contributing the most in each pairwise classification, six binary classifiers were trained across four conditions (See Section 2.5.2). We then defined the features shared by at least two binary classifiers with one common condition as the representative features of this condition. It follows that the features shared by the three classifiers can be interpreted as the most intrinsic neural integration for an experimental condition. For example, we defined the features selected via NCA when classifying O versus H, C versus H and H versus W as the representative features of class H.

#### 2.5.2 Classification Procedure

We selected the Support Vector Machine model to compare the performance of neural signatures of integration. According to a systematic literature review (Mari et al. 2022), SVM is one of the most common classifiers in the development of pain prediction models with EEG. We used an SVM with a linear kernel (kernel scale = 1; regularisation parameter *C* = 1, optimisation via the iterative single data algorithm, MATLAB's fitcsvm). Full specification of all hyperparameters, including the NCA regularisation parameter lambda and its selection procedure, is provided in Appendix A1.1. Six binary SVM classifiers (O vs. H, O vs. W, C vs. H, C vs. W, O vs. C and H vs. W) were trained on the selected features (Suykens and Vandewalle 1999). Then one dataset with 50% trials, excluded from the feature selection phase, was used as a dataset to quantify the performance of the classifiers. To avoid bias from unequal class sizes, we balanced the data in each binary classification by randomly selecting a subset of trials from the larger class to match the number of trials in the smaller class. The classifiers were then evaluated using 10‐fold cross‐validation, a standard approach for preventing overfitting and ensuring generalizability in machine learning with EEG data (Kohavi 1995; Varoquaux 2018). In summary, classifiers mainly discriminated tonic thermal pain (H), innocuous warm stimulation (W) and eyes‐open rest (O), whereas eyes‐closed (C) served as a sensory baseline; recursive feature selection identified the most informative integration metrics.

Within each evaluation half from Section 2.5.1, 10‐fold cross‐validation was conducted with folds assigned in temporal order, so that no epoch in a test fold shared time‐points with any epoch in the corresponding training folds. Class balancing was applied independently within each fold after the temporal partitioning.

Model performance was quantified using mean accuracy and per‐class accuracy across participants, with each class treated as the target class in turn. Because classifiers were trained and evaluated on class‐balanced datasets, per‐class accuracy for a given class is numerically equivalent to sensitivity for that class and to specificity when the other class is designated as positive; both metrics are therefore fully captured by the per‐class accuracy values reported in Table A1. To characterise estimation uncertainty, 95% confidence intervals were computed by bootstrap resampling (*N* = 5000 iterations) with replacement at the participant level, independently for each combination of classification pair, trial length and feature type. Bootstrap confidence intervals are reported alongside all accuracy estimates in Table A2.

#### 2.5.3 Statistical Inference

We used linear mixed models (LMM) as the primary inferential approach to account for repeated measurements within participants (Version 2.6.25, The Jamovi Project (2024), retrieved from https://www.jamovi.org). We used the 100 features selected by NCA in the analysis, with functional connectivity versus CFC, phase versus power and the pairwise comparisons across trial lengths (1, 2.5, 5 and 10 s) specified as fixed effects. In the pairwise classification of conditions (O vs. C, O vs. H, O vs. W, C vs. H, C vs. W and H vs. W), fixed effects included feature type (functional connectivity vs. CFC and phase vs. power) and trial length (1, 2.5, 5, 10 s), and the pairwise classification was modelled as random intercept:

$$\text{Accuracy}\sim {\text{feature type}}^{*}\;\text{trial length}+\left(1|\text{classification}\right)$$

The significance level (α) was set at 0.05, with the Bonferroni correction applied for post hoc testing. The confidence intervals were always 95%.

We analysed all pairwise contrasts (O vs. C, O vs. H, C vs. H, C vs. W, H vs. W and O vs. W). In particular, we pre‐specified eyes‐open state versus tonic thermal pain (O vs. H) as the primary contrast to identify integration signatures tied to painful states rather than to sensory stimulation alone, and treated H versus W as a secondary, sensory‐matched control to assess specificity. Primary inferential claims and feature‐selection summaries therefore emphasize O versus H, with H versus W reported in parallel when it corroborates or clarifies pain specificity. To further assess pain specificity, we conducted a cross‐condition transfer analysis in which classifiers trained on one condition pair were tested on a related pair sharing one condition (Appendix A5).

## 3 Results

### 3.1 Prediction Accuracy

**FIGURE 2:**
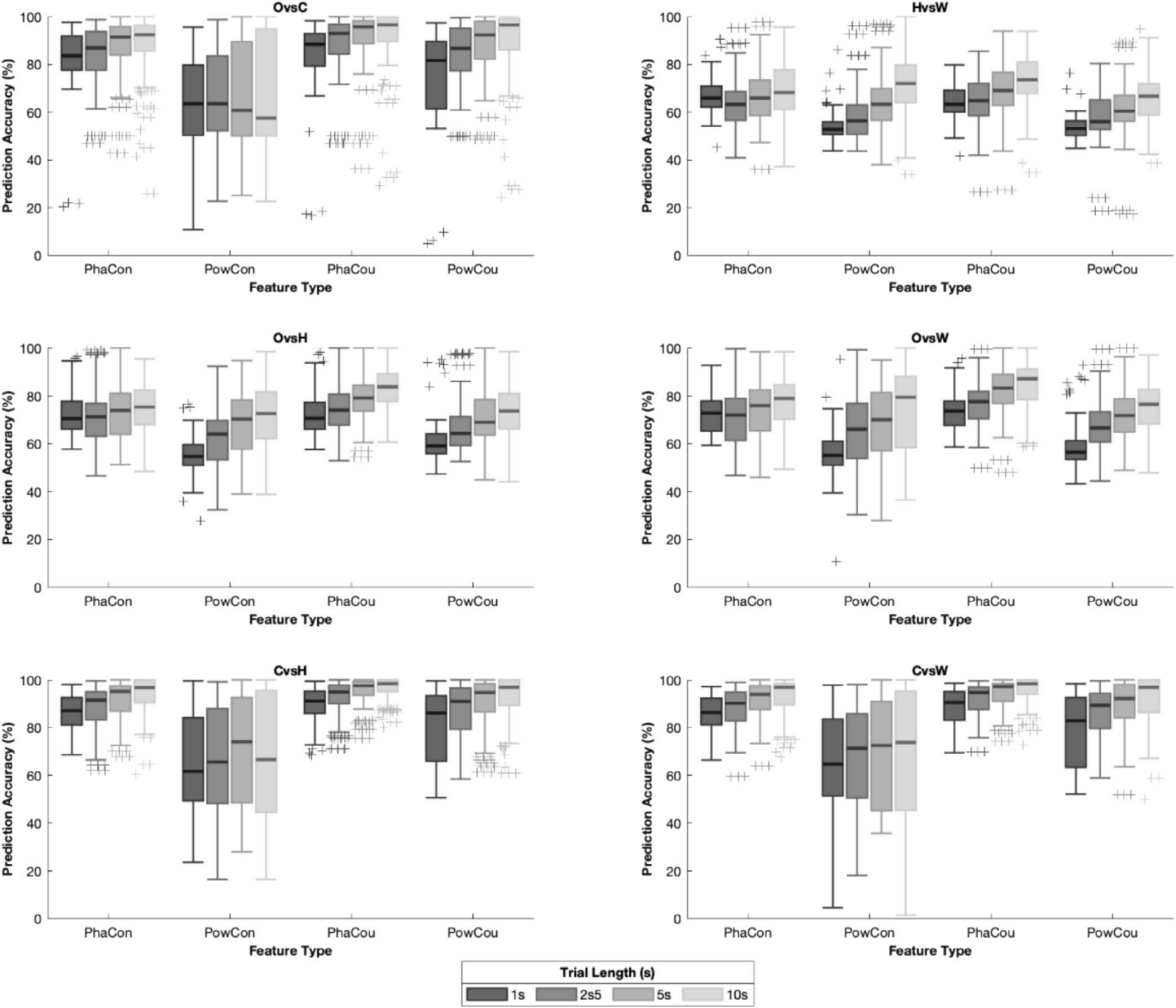
Boxplots of binary classification accuracy versus feature type. Each plot represents prediction accuracy (*y* axis) for the 100 features dataset across experimental conditions. The thick black line is the median accuracy, the box edges are the 25th and 75th percentiles, the outlines indicate the 95th percentile, the plus signs indicate values out of the confidence interval. The labels ‘O’ and ‘C’ represent ‘eyes‐open’ and ‘eyes‐closed’, while ‘H' and ‘W' stand for ‘hot’ and ‘warm’. Alpha‐phase connectivity (‘PhaCon’), alpha‐power connectivity (‘PowCon’), phase‐based CFC (‘PhaCou’) and power‐based CFC (‘PowCou’) are displayed on the *x* axis according to different trial lengths (arranged by different degrees of grey). Each subplot represents one pair of binary classification.

Phase‐based features consistently outperformed power‐based features in tonic thermal pain prediction. Table 1 displays the mean accuracy produced with alpha‐phase functional connectivity is 14.17% higher than alpha‐power connectivity ($t\left(9\right)=12.83$, ***p<0.001), and phase‐based CFC predicted pain with 7.12% higher mean accuracy than power‐based CFC ($t\left(9\right)=6.45$, ***p<0.001), there was no significant difference of the performances between alpha‐phase functional connectivity and phase‐based CFC (*p* = 0.058) (See all results in Table 1). LMM confirmed significant effects to accuracy of feature type ($F\left(3,9\right)=94.0,\;p<0.001$) and trial length ($F\left(3,9\right)=28.8,\;p<0.01$). Specific to trial length, classification accuracy increased monotonically with trial length (68.9% at 1 s, 75.2% at 2.5 s, 79.1% at 5 s and 81.3% at 10 s, see Figure 2). Consistent with our a priori emphasis, the primary analyses focused on the eyes‐open (O) versus hot (H) contrast; then H versus W comparisons were conducted to verify whether signatures that discriminate pain from rest also distinguish painful from innocuous thermal stimulation. Across models, the effects were significant for O versus H (feature type: $F\left(3,9\right)=18.3,\;p<0.001$; trial length: $F\left(3,9\right)=13.0,\;p<0.01$) and H versus W (feature type: $F\left(3,9\right)=8.4,\;p<0.01$; trial length: $F\left(3,9\right)=12.6,\;p<0.01$).

**TABLE 1:** Summary of post hoc pairwise comparisons of prediction accuracy across factor levels. Factors include trial length (1, 2.5, 5, 10 s) and feature type (PhaCon, PhaCou, PowCon, PowCou). Asterisks indicate statistical significance after Bonferroni correction (*< 0.05, **< 0.01, ***< 0.001).

	Factor 1	Factor 2	Pairwise comparison between factor levels				
			Difference	SE	*t*	df	*p*
(a) Trial length	10	5	0.0253	0.0110	2.05	9	0.263
	10	2.5	0.0596	0.0110	4.83	9	***< 0.001
	10	1	0.0964	0.0110	7.81	9	***< 0.001
	5	2.5	0.0343	0.0110	2.78	9	0.041
	5	1	0.0711	0.0110	5.76	9	***< 0.001
	2.5	1	0.0368	0.0110	2.98	9	0.041
(b) Feature type	PhaCou	PhaCon	0.0328	0.0110	2.66	9	0.058
	PhaCou	PowCou	0.0712	0.0110	5.77	9	***< 0.001
	PhaCou	PowCon	0.1744	0.0110	14.14	9	***< 0.001
	PhaCon	PowCon	0.1417	0.0110	11.48	9	***< 0.001
	PhaCon	PowCou	0.0384	0.0110	3.12	9	*0.016
	PowCou	PowCon	0.1032	0.0110	8.37	9	***< 0.001

**FIGURE 3:**
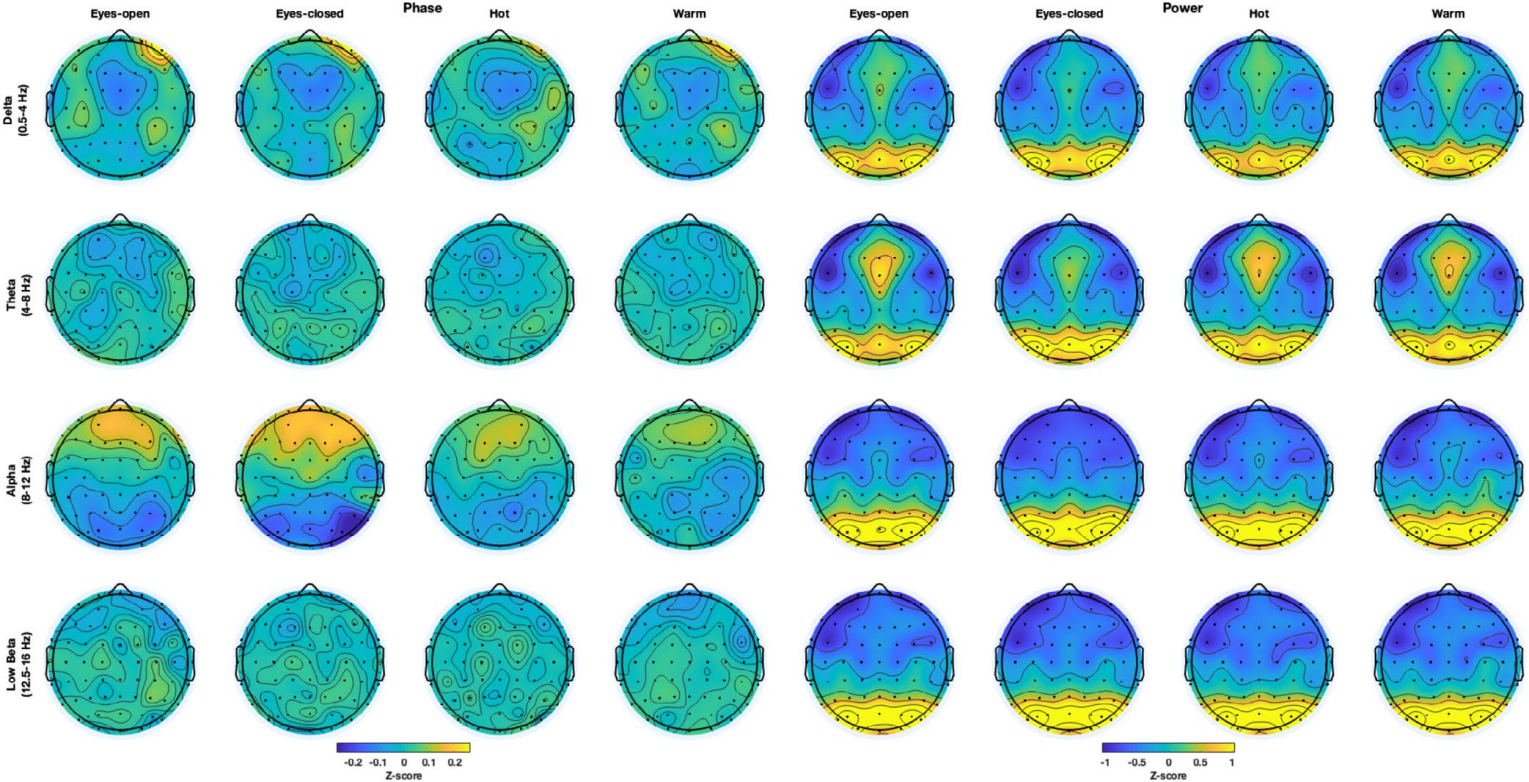
Brain topography of *z*‐scored exponential phases (Phase) and amplitudes (Power) versus conditions and frequency bands. In the subplots of phase, each plot represents the *z*‐scored exponential phases at each electrode. In the subplots of power, each plot represents the z‐scored brain topography of the median signal amplitudes calculated as the square root of power spectral density (PSD). Each row of plots represents the topography from the same frequency band, and each column of plots represents the same experimental condition.

Considering that the only difference between spectral coherence and ISPC is the presence of the signal amplitudes, these comparisons suggest that the introduction of power‐based information weakened the performance of the pain prediction model. Therefore, we analysed KLD between conditions using the topography of magnitudes or exponential phases as mentioned in Section 2.4.3. Figure 3 shows the power‐ and phase‐based connectivity topography with the mean values across all samples of different pairwise classifications within different frequency bands. We observed that phase information carries broader differences across conditions than power. Figure A6 shows how within each frequency band (delta, theta, alpha, low beta), the rescaled KLD of magnitude was 0.2298±0.1593, whereas the rescaled KLD of exponential phase was 0.9407±0.0185. Paired t‐tests across these contrasts confirmed that the KLDs of phase values were significantly larger than the KLDs of magnitudes ($t\left(46\right)=38.97,\;p<0.001$).

### 3.2 Feature Visualisation

**FIGURE 4:**
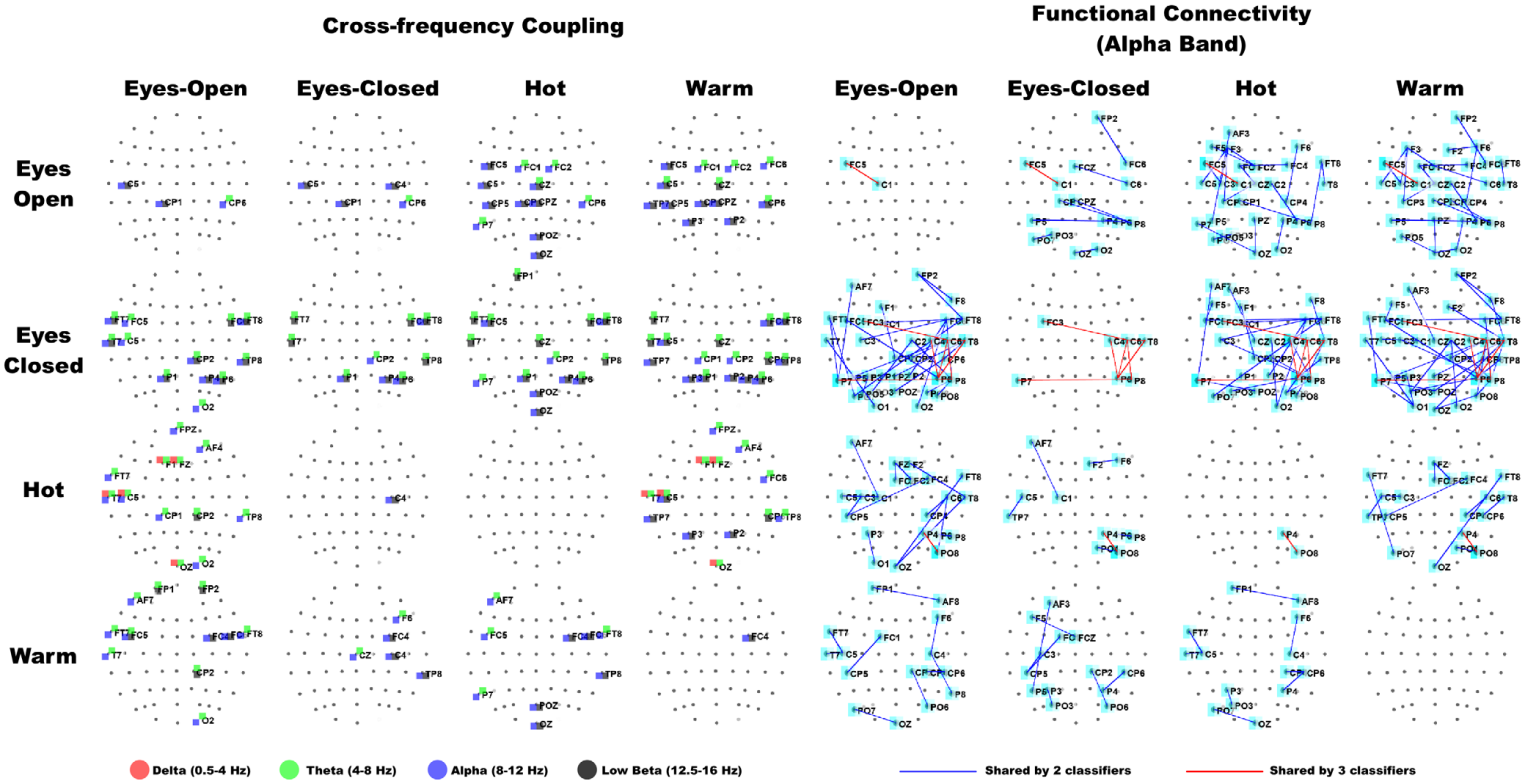
Topographical distribution of phase‐based neural signatures. Except for the diagonal from top‐left to right‐bottom, the row represents one condition, the column represents the other condition in the binary classification. (1) In the cross‐frequency coupling patterns, the involved frequency bands are marked in different colours, which are presented in the legend. (2) In the functional connectivity plots (all alpha band), features that are shared by at least two classifiers were marked with blue lines. Features shared by all classifiers of a particular row were marked in red (only these features were marked on the diagonal).

Figure 4 highlights that frontal and occipital areas emerged as the main regions contributing to pain classification. Interestingly, many features that distinguish the pain condition (H) from eyes‐open (O) also appear in the H versus W comparison. This overlap is consistent with these features relating specifically to pain rather than to general differences between resting and thermal stimulation states.

Concerning the CFC features, pain‐related integration was primarily located in the frontal region, including theta–alpha coupling at FPz and AF4 and delta–theta coupling at Fz and F1. By contrast, CFC patterns more characteristic of the non‐painful conditions (O, C and W) were observed in occipital regions, such as alpha–beta coupling at POz and Oz.

Concerning the functional connectivity features, short‐range frontal connections (e.g., FP2–AF8, Fz–FC2) and long‐range connections linking frontal and occipital sites (FT8–Oz) contributed to pain prediction. Additional connections between occipital and parietal regions (e.g., P4–PO8) also showed involvement.

## 4 Discussion

The present study contributes to the pain literature in three ways. First, by highlighting which aspects of EEG connectivity can be most informative of pain ‐related brain states. In comparing inter‐site phase clustering with magnitude‐squared coherence within the same dataset, we show that the pain‐related informativeness of alpha‐band coherence is driven by phase synchrony rather than amplitude covariation. Second, the multivariate classification framework evaluates the joint discriminative value of connectivity and coupling features, extending earlier correlational findings to within‐participant pain‐state discrimination. Third, NCA‐derived feature weights identify which specific connections and frequency couplings are most informative, providing a physiologically interpretable account of tonic pain‐related neural integration. Thus, the contribution of the study is not simply that alpha coherence is related to pain, but that it specifies what aspect of coherence is pain‐relevant and how that information is organised across integration features.

### 4.1 From Data‐Driven Findings to Interpretation of Optimal EEG Measures of Neural Integration

Previous studies have highlighted the importance of alpha activity and neural synchronisation in pain perception. For example, several studies reported correlations between alpha power, peak alpha frequency and pain intensity (Furman et al. 2018; Nir et al. 2012, 2010; Valentini et al. 2022) and other work has shown distinct desynchronisation patterns in lower (7–10 Hz) and upper (10–12 Hz) alpha bands across pain, non‐painful and psychologically unpleasant conditions (Klimesch 1999; Nir et al. 2012). More directly relevant to the present study, alpha‐phase synchrony has been directly implicated in pain‐related processing; Chowdhury et al. (2025) reported changes in alpha‐band phase coherence in frontal and occipital regions that correlated with individual pain thresholds during experimental heat pain, a topographic pattern that closely overlaps with the signatures we identify below. Reductions in alpha‐phase coherence have also been reported in chronic pain populations, including fibromyalgia (Jakobsen et al. 2026) and mixed‐aetiology chronic pain (De Martino et al. 2025), though whether tonic experimental pain in healthy individuals and chronic pain share the same neural mechanisms remains an open question. While many machine learning approaches to pain prediction have emerged (Mari et al. 2022), several combined multiple features, making physiological interpretation difficult. The present study addresses this gap by directly comparing phase‐ and power‐based integration features, using classification accuracy as a principled index of their relative informativeness, and using feature selection weights to identify which specific connections and frequency couplings are engaged during tonic pain.

In this study, candidate features of neural integration were derived from both functional connectivity and CFC. Our results showed that phase‐based features from both domains consistently outperformed power‐based features in predicting pain. As demonstrated in Formulas (1) and (4), magnitude‐squared coherence can be expressed as ISPC weighted by signal amplitude, meaning that the inclusion of power information reduced condition‐specific discriminability. This was confirmed by topographical analyses (Figure 3), which revealed that power distributions were more similar across conditions, whereas phase‐based features preserved meaningful distinctions. Accordingly, widespread alpha‐phase connectivity was observed (Figure 4), underscoring the role of synchronisation processes in pain prediction. These findings go beyond confirming that alpha‐phase coherence is associated with pain (Chowdhury et al. 2025). By isolating the phase component from amplitude, we demonstrate that the pain‐specificity of coherence is driven by phase synchrony rather than power co‐fluctuation. Furthermore, the selected features reflect pain‐specific neural activity rather than artefactual signal; the topographic results distinguish brain regions whose pain‐related activity is phase‐ versus power‐driven, and the framework demonstrated that ML‐based feature selection can yield physiologically interpretable signatures of pain.

Finally, our analysis also addressed methodological considerations regarding trial length. Longer trial segments (up to 10 s) yielded higher classification accuracy than shorter ones, despite reducing the number of available samples. Because longer epochs yield fewer trials from the same fixed‐duration recording, this improvement cannot be attributed to a larger training set; rather, it reflects more precise connectivity estimates afforded by a greater number of oscillatory cycles available for phase‐based calculations. This pattern is consistent with prior observations that connectivity measures stabilise only in longer epochs (Fraschini et al. 2016), and it underscores the importance of carefully balancing trial duration with feasibility when designing EEG‐based pain prediction paradigms.

### 4.2 Topographical Characterisation of Neural Integration During Tonic Pain

The topographical patterns in Figure 4 highlight that pain‐related neural signatures are not uniformly distributed but instead involve specific large‐scale integration patterns. Some work revealed that frontal alpha activity is key in pain experience. For example, Mayaud et al. (2019) reported that alpha‐phase synchrony tracked changes in low back pain, and Camfferman et al. (2017) observed that frontal alpha power reflects chronic pain states. Rustamov et al. (2021, 2022) suggested that the enhancement of frontal theta power is a signature of tonic pain recovery, a feature shared by both experimental tonic and chronic pain. To our knowledge, frontal delta‐band involvement in tonic pain processing is understudied. Huber et al. (2006) reported a brain‐wide increase in delta power specific to tonic thermal pain, but delta‐frequency phase coupling within frontal networks has not previously been characterised in this context. Our identification of delta‐theta CFC at frontal sites (i.e., Fz, F1) as a pain‐specific signature thus extends the existing literature beyond power‐based delta observations towards a connectivity‐level account.

Our analysis revealed that alpha‐band activity in frontal regions was central for predicting pain in contrast to non‐painful conditions. Specifically, theta–alpha and delta‐theta frontal coupling and short‐range alpha frontal connectivity emerged as key signatures. These findings are in line with earlier evidence linking frontal alpha activity with pain experience.

Stam et al. (2007) indicated that the occipital synchrony and occipital‐frontal interactions strengthen during resting states. Hu et al. (2013) suggested alpha event‐related desynchronisation in parietal and occipital regions is a marker of pain perception. We also found that occipital involvement, particularly in alpha‐phase connectivity, is key to distinguishing pain from resting states. It suggested that the change of occipital‐frontal connectivity is specific to pain states. Together, these results disclosed the role of frontal and occipital alpha‐band activity as central components of pain‐related neural signatures.

In addition, the somatosensory cortex activity was suggested to be associated with pain sensitivity and intensity (Riedl et al. 2011; Spisak et al. 2020; Tu et al. 2019). It may contribute to separating pain‐specific from cognitive‐aspecific components. Features involving central electrodes suggested that, assuming central scalp activity can be interpreted as an index of somatosensory cortex processing, the somatosensory‐related processes may have participated in both pain processing and sensory perception. In our analysis, CFC features involving somatosensory sites were most evident when distinguishing thermal stimulation (H or W) from resting states (O or C). For example, alpha‐phase connectivity between C1 and AF7 differentiated pain (H) from rest (O), whereas FC1–CP5 and CP2–PO6 differentiated W from rest. By contrast, features distinguishing O from C were more spatially restricted, clustered around somatosensory and nearby regions. To verify that these models capture pain‐specific signatures, we conducted a cross‐condition evaluation (Appendix A5), which confirmed that features discriminating H from W and from resting states reflect pain‐specific neural integration rather than general thermal or arousal‐related processing. These observations align with the somatosensory–prefrontal network proposed for tonic experimental pain by Nickel et al. (2020), supporting its relevance for future applications.

### 4.3 Limitations

Several limitations warrant consideration. First, the sample is demographically homogeneous (predominantly white/Caucasian, young healthy adults), which limits its generalisation to e.g., elderly, clinical pain populations or individuals with neurological or psychiatric conditions. Second, machine learning analyses were conducted within participants. Cross‐subject generalisation of the SVM model on the same dataset was below chance level (Han 2024), highlighting inter‐individual variability in EEG connectivity patterns as a key challenge in future work. To address this challenge, we developed a convolutional neural network (CNN) model using the same alpha‐phase connectivity features, which improved the cross‐subject accuracy from below chance (SVM: 37.24%) to above chance (CNN: 63.69%; Han 2024; Han et al. 2023). Third, and most fundamentally, the findings should be understood as identifying candidate neural‐integration signatures of tonic experimental pain in healthy individuals rather than fully applicable clinical biomarkers. Accordingly, generalisation metrics such as cross‐condition transfer are outside of the scope of this comparative framework. Alpha‐band activity has also been investigated in chronic pain, but findings are highly inconsistent across studies. Tonic experimental pain and chronic clinical pain differ not only in duration and aetiology but in their neural spectral profiles: resting‐state EEG studies of chronic and neuropathic pain consistently implicate theta‐band increases and alpha decreases, whereas gamma increases prominent in experimental pain are absent in neuropathic pain (Bott et al. 2025; Mussigmann et al. 2022; Zebhauser et al. 2023). These divergences suggest that the phase‐based alpha signatures identified here reflect mechanisms of acute nociceptive processing, that is, sensory gating and transient large‐scale synchronisation that are distinct from the thalamocortical reorganisation associated with chronic pain. Direct translation to clinical populations therefore requires dedicated investigation in patient cohorts, and the present study is best understood as a necessary prior step: establishing which feature class best captures the tonic pain state before generalisation can be meaningfully pursued.

## 5 Conclusions

This study used EEG‐based measures and machine learning to investigate neural signatures of integration during experimental tonic pain. Phase‐based synchrony emerged as the most reliable predictor, distinguishing pain from non‐painful states with key contributions from frontal, occipital and somatosensory regions. With phase‐based synchrony between brain regions or frequency bands, alpha‐band connectivity and CFC involving theta, alpha and low beta bands were particularly informative. Longer trial lengths improved prediction accuracy by enhancing the stability of integration measures.

Together, these findings provide a data‐driven framework for identifying physiologically interpretable EEG signatures of pain. Beyond advancing mechanistic understanding, such signatures may support the development of objective pain assessment tools, which could complement self‐report in both research and clinical settings.

## Author Contributions

Y.H. was responsible for methodology development, formal analysis, visualisation and writing of the original draft. E.V. contributed to conceptualisation, methodology and investigation, and was responsible for project administration, supervision and review and editing of the manuscript. S.H. contributed to conceptualisation, provided supervision and participated in review and editing. All authors discussed the results, provided critical feedback and approved the final version of the manuscript.

## Funding

S.H. was supported by UKRI EPSRC (UKRI824: PaiNeuro: Towards objective PAIn measurement using NEUROphysiological signals). Y.H. was awarded with Faculty of Science and Health Studentship from the University of Essex. The Jeio Tech RW‐0525P water bath was purchased by means of a Departmental Research Promotion and Impact Fund award to E.V.

## Conflicts of Interest

The authors declare no conflicts of interest.

## Supporting information


**Appendix A1.** Signal processing and machine learning: formulae and hyperparameters.
**Appendix A1.1.** Current source density (CSD).
**Appendix A1.2.** Kullback‐Leibler divergence.
**Appendix A1.3.** Neighbourhood component analysis.
**Appendix A2.** Feature number of prediction accuracy.
**Appendix A2.1.** Methods.
**A2.2.** Results.
**Figure A1.** Prediction accuracy as a function of the number of features. Accuracy increased with more features and plateaued after approximately 60–80 features in all binary classifications except for H versus W. Results are averaged across all trial lengths and feature types.
**Figure A2.** Topographical distribution of dwPLI‐based neural signatures. Except for the diagonal from top‐left to right‐bottom, the row represents one condition, the column represents the other condition in the binary classification. (1) In the cross‐frequency coupling patterns, the involved frequency bands are marked in different colours, which are presented in the legend. (2) In the functional connectivity, features that are shared by at least two classifiers were marked with blue lines. Features shared by all classifiers of a particular row were marked in red (only these features were marked on the diagonal).
**Figure A3.** An example schematic of the cross‐condition transfer analysis. Left: The HOT thermal pain condition (H) shares overlapping sensory components with two non‐painful control conditions: Warm thermal innocuous (W), which shares both thermal stimulation and visual cognition, and Eyes‐open resting state (O), which shares only visual input. Right: Transfer design between the two classification pairs. A classifier trained on H versus W was tested on H versus O, and vice versa. Transfer from H versus W to H versus O preserved classification performance (indicating that the H vs. W model captures pain‐specific signatures), whereas transfer from H versus O to H versus W significantly degraded performance (indicating that the H vs. O model relies more heavily on thermal sensation features versus visual cognition).
**Figure A4.** Cross‐condition transfer accuracy for binary classification. Cell colour indicates the Bonferroni‐corrected significance of paired t‐tests comparing transfer accuracy to standard (within‐condition) accuracy. *t*‐values are displayed within each cell, where positive values indicate transfer accuracy exceeding standard accuracy, and negative values indicate the converse. Rows correspond to feature types and columns to transfer directions (training condition→testing condition).
**Figure A5.** Confusion matrices of binary classifications of interest from 10‐s trials. There are two types of classifications of interest, including pain (H) versus non‐painful (O/W) and resting state (O) versus thermal stimuli (H/W). Each figure involves the confusion matrices produced by different neural signatures, where ‘Conn’ represents functional connectivity, and ‘Coup’ represents cross‐frequency coupling (CFC). Totally, phase‐based CFC was the best signature in all classifications of interest.
**Figure A6.** Heatmaps illustrating the mean Kullback–Leibler divergence (KLD) for *z*‐scored exponential phases (Phase) and magnitude (Power) distributions. The KLDs were produced across four frequency bands (Delta, Theta, Alpha, Beta) and six condition comparisons (O vs. C, O vs. H, O vs. W, C vs. H, C vs. W, H vs. W). Each cell shows the mean KLD value across 62 channels x 36 participants for the corresponding frequency band and condition contrast. Darker shades denote higher divergence (i.e., greater discrepancy) between the compared distributions. For observation, the KLDs were rescaled between 0 and 1 according to the maximum and minimum KLDs from both measures.
**Table A1.** Accuracy of different classifications, feature types and trial lengths for pain prediction. Accuracy values are reported for six binary classifications (C vs. H, C vs. W, H vs. W, O vs. C, O vs. H, O vs. W) across four EEG‐based feature types: Phase‐based Functional Connectivity (PhaCon), Phase‐based Cross‐frequency Coupling (PhaCou), Power‐based Functional Connectivity (PowCon) and Power‐based Cross‐frequency Coupling (PowCou). Each feature type was evaluated at four trial lengths (1 s, 2.5 s, 5 s and 10 s). Values correspond to mean classification performance across 36 participants. Bold values indicate the main comparisons, that is, selected trial length, classification pairs, and feature types. The accuracy of each class is the model's sensitivity when we define the class as the true label, for example, when we define H as the true label in H versus W, the accuracy of H is the sensitivity, and the accuracy of W is its specificity.
**Table A2.** Mean classification accuracy with 95% bootstrap confidence intervals across feature types, trial lengths and pain state classification pairs. Each cell reports the mean accuracy [95% CI lower bound, 95% CI upper bound] estimated via bootstrap resampling (5000 iterations) across cross‐validation folds. Sub‐column letters indicate the class for which sensitivity is reported. Bold italic values denote the HvsW and OvsH classification pairs at the 10 s trial length.
**Table A3.** Mean classification accuracy ± their standard deviation of dwPLI‐based features in percentage.
**Table A4.** Summary of post hoc pairwise comparisons of prediction accuracy across feature types. The post hoc comparisons including non‐dwPLI features are ignored in this table, which are equivalent with the results in Table 1. dwPLICon represents dwPLI‐based functional connectivity at the alpha band, and dwPLICou represents the dwPLI‐based cross‐frequency coupling across delta, theta, alpha and low beta bands.
**Table A5.** Cross‐condition transfer accuracy for four feature types. Each row reports one transfer direction (e.g., HW_to_HO denotes a model trained on H vs. W and tested on H vs. O). Transfer accuracy (mean ± standard deviation) is compared against the standard within‐classification accuracy of the training conditions, matched the same 36 participants, Bonferroni‐corrected *p*‐values are reported (PhaseConn, alpha‐phase functional connectivity; PowerConn, power‐based function connectivity; PhaseCoup, alpha‐phase CFC; PowerCoup, power‐based CFC).

## Data Availability

All analysis code (preprocessing, feature extraction, NCA feature selection, SVM classification and visualisation) is publicly available at https://github.com/han‐yy/NeuralMarkerPain. EEG data are available at https://osf.io/2mqtb/files/osfstorage.
