# Supplementary material for: Predicting Pain: Electroencephalography Signatures of Neural Integration During Experimental Tonic Thermal Pain
Source: Eur J Pain. 2026 Jun 30;30(6):e70313. doi: 10.1002/ejp.70313 (PMC13317760; doi:10.1002/ejp.70313)
Supplement: Supplementary file 1 — Appendix A1. Signal processing and machine learning: formulae and hyperparameters. Appendix A1.1. Current source density (CSD). Appendix A1.2. Kullback‐Leibler divergence. Appendix A1.3. Neighbourhood component analysis. Appendix A2. Feature number of prediction accuracy. Appendix A2.1. Methods. A2.2. Results. Figure A1. Prediction accuracy as a function of the number of features. Accuracy increased with more features and plateaued after approximately 60–80 features in all binary classifications except for H versus W. Results are averaged across all trial lengths and feature types. Figure A2. Topographical distribution of dwPLI‐based neural signatures. Except for the diagonal from top‐left to right‐bottom, the row represents one condition, the column represents the other condition in the binary classification. (1) In the cross‐frequency coupling patterns, the involved frequency bands are marked in different colours, which are presented in the legend. (2) In the functional connectivity, features that are shared by at least two classifiers were marked with blue lines. Features shared by all classifiers of a particular row were marked in red (only these features were marked on the diagonal). Figure A3. An example schematic of the cross‐condition transfer analysis. Left: The HOT thermal pain condition (H) shares overlapping sensory components with two non‐painful control conditions: Warm thermal innocuous (W), which shares both thermal stimulation and visual cognition, and Eyes‐open resting state (O), which shares only visual input. Right: Transfer design between the two classification pairs. A classifier trained on H versus W was tested on H versus O, and vice versa. Transfer from H versus W to H versus O preserved classification performance (indicating that the H vs. W model captures pain‐specific signatures), whereas transfer from H versus O to H versus W significantly degraded performance (indicating that the H vs. O model relies more heavily on thermal sensation fe [file EJP-30-0-s001.docx]

**Appendix**

**A1 Signal Processing and Machine Learning: Formulae and Hyperparameters**

**A1.1 Current Source Density (CSD)**

Mathematically, the CSD relies on the Laplacian transform, i.e., the second spatial derivative of the electric potential from each EEG channel. In this work, the Laplacian at a channel $C$ was approximated as:

$$\begin{aligned} \Delta^{2}V_{C}=4V_{C}-V_{\text{left}}-V_{\text{right}}-V_{\text{up}}-V_{\text{down}} \#\left( A1 \right) \end{aligned}$$

where $\Delta^{2}V(x,y)$ is the Laplacian of voltage $V$ at the given point $(x,y)$.

**A1.2 Kullback-Leibler Divergence**

The Kullback-Leibler divergence (KLD) between two probability distributions $P$ and $Q$ over a random variable $X$ (which can represent either amplitudes or exponential phases) is defined as (Kullback & Leibler, 1951):

$$\begin{aligned} D_{KL}\left( P\parallel Q \right)=\sum_{x\mathcal{\in X}} P\left( x \right)\log\frac{P\left( x \right)}{Q\left( x \right)} \#\left( A2 \right) \end{aligned}$$

In our study, for each participant we computed the KLD between the distributions $P$ and $Q$ at the same channel from each participant, where $X$ denotes the measured amplitudes (or exponential phases). The resulting value quantifies how much the two distributions differ at that channel. For comparisons between groups or conditions, we averaged the KLD values across all channels:

$$\begin{aligned} \overline{D}_{KL}=\frac{1}{M}\sum_{s=1}^{M} \frac{1}{N}\sum_{c=1}^{N} D_{KL}^{\left( sc \right)} \text{with} D_{KL}^{\left( sc \right)}=\sum_{x\mathcal{\in X}} P_{sc}\left( x \right)\log\frac{P_{sc}\left( x \right)}{Q_{sc}\left( x \right)}\#\left( A3 \right) \end{aligned}$$

where $N$ is the total number of channels, and $P_{c}$ and $Q_{c}$ represent the distributions of $X$ at channel $c$ from participant $s$ for the two classes being compared.

This approach follows the original formulation of KLD and is widely used in signal processing and neuroscience applications (Cover, 1999; Mancini et al., 2022; Ryu et al., 2024). Considering the range of KLD is from 0 to infinity, we rescaled the KLDs according to the minimum and maximum KLDs from the union of two KLD sets. Hence, the rescaled KLDs are between 0 and 1.

**A1.3 Neighbourhood Component Analysis**

To optimally select the important features, we used NCA, a supervised learning method for offline feature selection. NCA asks for samples independent of the classifier training using the following approach (Yang et al., 2012). If the distance between two samples in a dataset $T=(\mathbf{x}_{\mathbf{i}},y_{i}),i\in[1,N],\mathbf{x}_{\mathbf{i}}\in R^{d}$ containing $N$ labelled samples, where $\mathbf{x}_{\mathbf{i}}$ are the feature vectors of samples, and $y_{i}\in\{1,2,\ldots,c\}$ are the class labels representing the classes with the total number of $c$ in the classification (in this study, $c=4$), then the distance between two samples $\mathbf{x}_{\mathbf{i}}$ and $\mathbf{x}_{\mathbf{j}}$ is defined as:

$$\begin{aligned} \begin{matrix} D_{w}\left( \mathbf{x}_{\mathbf{i}},\mathbf{x}_{\mathbf{j}} \right)=\sum_{l=1}^{d} w_{l}^{2}\left| x_{il}-x_{jl} \right| \end{matrix} \#\left( A4 \right) \end{aligned}$$

NCA aims to optimise the weights $w_{l}$ to maximise the accuracy of the leave-one-out nearest neighbour (NN) classification. The final weights would be referred to as:

$$\begin{aligned} \begin{matrix} \mathbf{w}=\mathrm{argmax}_{w_{l}}\left( \sum_{i} \sum_{j} y_{ij}p_{ij}-\lambda\sum_{l=1}^{d} w_{l}^{2} \right) \end{matrix} \#\left( A5 \right) \end{aligned}$$

where $p_{ij}$ is the joint probability of samples $\mathbf{x}_{\mathbf{i}}$ and $\mathbf{x}_{\mathbf{j}}$, ($y_{ij}=1$ when $y_{i}=y_{j}$. $y_{ij}=0$), $\lambda$ is a regularisation parameter tuned through cross-validation. The output $\mathbf{w}$ is the optimised weight vector that represents the significance of each feature. According to the preliminary analysis in the Appendix, we then selected 100 features with the highest weights $\mathbf{w}$ within each binary classification.

**A2 Feature Number of Prediction Accuracy**

**A2.1 Methods**

To investigate the impact of the number of features on prediction accuracy, we employed neighbourhood component analysis (NCA) for feature selection and support vector machine (SVM) classifiers for evaluation (Section 2.5). From each participant, the top-ranked features were determined according to their NCA-derived weights. The main analyses were performed with 100 features, chosen to provide a robust reference set that captures the most informative aspects of the data while minimizing the risk of underfitting. Additional analyses were carried out with subsets of 10, 20, 30, ..., 90, and 150, 200 features, ranked by their cumulative importance across participants. Classification was performed across all trial lengths and feature types, and prediction accuracy was averaged accordingly. This procedure enabled a systematic assessment of how classifier performance scales with the number of features.

**A2.2 Results**

Prediction accuracy increased as the number of features grew, but the improvement was not linear. When fewer than 20 features were included, classification accuracy remained unstable and clearly below the performance obtained with larger sets. As the feature number increased to between 30 and 60, accuracy improved substantially and approached the level of the 100-feature reference model. Beyond this range, the benefits of additional features diminished in all binary classifications except for H vs W, which showed a modest continued improvements up to 200 features. Because the primary aim of this study is comparative rather than optimisation of absolute accuracy, and because the relative ordering between feature categories remains consistent across the full 10-200 feature range, the choice of 100 features provides a stable and representative benchmark for the comparative analysis. Further increases beyond 100 features resulted only in marginal gains, indicating that most additional features contributed redundancy rather than new discriminative information.


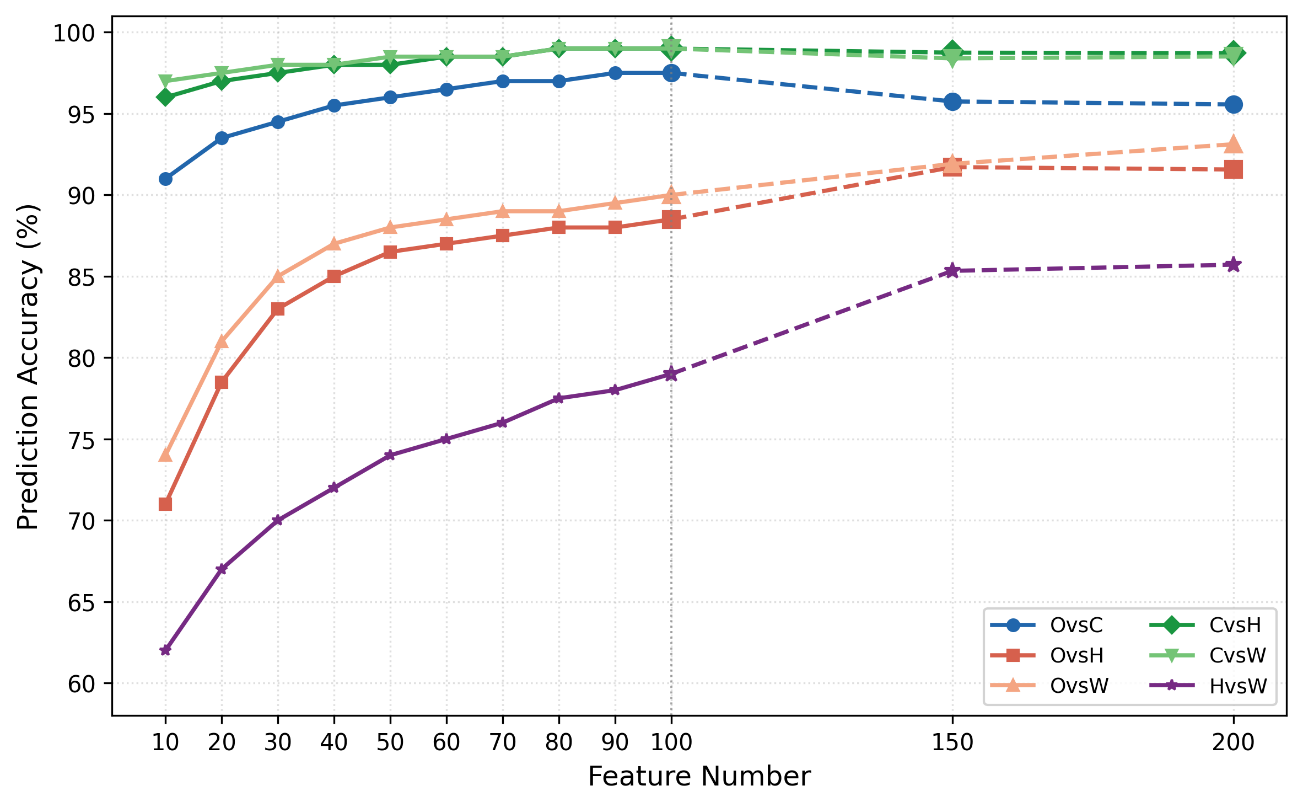


**Figure A1.** Prediction accuracy as a function of the number of features. Accuracy increased with more features and plateaued after approximately 60--80 features in all binary classifications except for H vs W. Results are averaged across all trial lengths and feature types.

**A3 Sensitivity Analysis of Classifications, Feature Types, and Trial Lengths**

**Table A1.** **Accuracy of different classifications, feature types, and trial lengths for pain prediction.** Accuracy values are reported for six binary classifications (CvsH, CvsW, HvsW, OvsC, OvsH, OvsW) across four EEG-based feature types: Phase-based Functional Connectivity (PhaCon), Phase-based Cross-frequency Coupling (PhaCou), Power-based Functional Connectivity (PowCon), and Power-based Cross-frequency Coupling (PowCou). Each feature type was evaluated at four trial lengths (1s, 2.5s, 5s, and 10s). Values correspond to mean classification performance across 36 participants. Bold values indicate the main comparisons, i.e., selected trial length, classification pairs, and feature types. The accuracy of each class is the model’s sensitivity when we define the class as the true label, for example, when we define H as the true label in H vs. W, the accuracy of H is the sensitivity, and the accuracy of W is its specificity.

**Table A2.** **Mean classification accuracy with 95% bootstrap confidence intervals across feature types, trial lengths, and pain state classification pairs.** Each cell reports the mean accuracy [95% CI lower bound, 95% CI upper bound] estimated via bootstrap resampling (5,000 iterations) across cross-validation folds. Sub-column letters indicate the class for which sensitivity is reported. Bold italic values denote the HvsW and OvsH classification pairs at the 10 s trial length.

|  |  | **Classification** | | | | | |
| --- | --- | --- | --- | --- | --- | --- | --- |
| **Feature Type** | **Trial Length** | CvsH | CvsW | HvsW | OvsC | OvsH | OvsW |
| **PhaCon** | 1s | 0.8625 [0.8439, 0.8809] | 0.8594 [0.8417, 0.8763] | 0.6685 [0.6506, 0.6864] | 0.8172 [0.7806, 0.8491] | 0.7250 [0.7050, 0.7466] | 0.7258 [0.7065, 0.7462] |
|  | 2.5s | 0.8859 [0.8739, 0.8979] | 0.8815 [0.8698, 0.8926] | 0.6423 [0.6287, 0.6566] | 0.8420 [0.8248, 0.8583] | 0.7097 [0.6942, 0.7252] | 0.7111 [0.6945, 0.7277] |
|  | 5s | 0.9149 [0.9033, 0.9257] | 0.9127 [0.9021, 0.9232] | 0.6685 [0.6525, 0.6847] | 0.8729 [0.8556, 0.8892] | 0.7417 [0.7260, 0.7586] | 0.7472 [0.7309, 0.7632] |
|  | **10s** | 0.9390 [0.9291, 0.9483] | 0.9357 [0.9264, 0.9452] | ***0.6939 [0.6783, 0.7100]*** | 0.8844 [0.8660, 0.9009] | ***0.7542 [0.7401, 0.7686]*** | 0.7750 [0.7602, 0.7894] |
| **PhaCou** | 1s | 0.8909 [0.8720, 0.9096] | 0.8857 [0.8676, 0.9028] | 0.6408 [0.6248, 0.6568] | 0.8370 [0.7974, 0.8711] | 0.7222 [0.7019, 0.7436] | 0.7368 [0.7172, 0.7563] |
|  | 2.5s | 0.9263 [0.9161, 0.9359] | 0.9181 [0.9080, 0.9279] | 0.6436 [0.6290, 0.6577] | 0.8811 [0.8646, 0.8976] | 0.7467 [0.7322, 0.7614] | 0.7653 [0.7517, 0.7785] |
|  | 5s | 0.9520 [0.9440, 0.9594] | 0.9435 [0.9350, 0.9513] | 0.6901 [0.6748, 0.7049] | 0.9087 [0.8915, 0.9253] | 0.7957 [0.7826, 0.8086] | 0.8182 [0.8038, 0.8319] |
|  | **10s** | 0.9662 [0.9604, 0.9721] | 0.9585 [0.9515, 0.9651] | ***0.7344 [0.7198, 0.7484]*** | 0.9209 [0.9037, 0.9367] | ***0.8298 [0.8180, 0.8412]*** | 0.8454 [0.8329, 0.8578] |
| **PowCon** | 1s | 0.6466 [0.5972, 0.6953] | 0.6582 [0.6105, 0.7064] | 0.5430 [0.5283, 0.5587] | 0.6333 [0.5842, 0.6800] | 0.5479 [0.5290, 0.5661] | 0.5651 [0.5409, 0.5897] |
|  | 2.5s | 0.6616 [0.6317, 0.6912] | 0.6805 [0.6509, 0.7089] | 0.5858 [0.5717, 0.6007] | 0.6653 [0.6404, 0.6903] | 0.6265 [0.6096, 0.6428] | 0.6513 [0.6318, 0.6708] |
|  | 5s | 0.6974 [0.6663, 0.7274] | 0.6888 [0.6594, 0.7179] | 0.6411 [0.6255, 0.6573] | 0.6720 [0.6434, 0.6994] | 0.6892 [0.6722, 0.7069] | 0.6841 [0.6623, 0.7047] |
|  | **10s** | 0.6925 [0.6606, 0.7246] | 0.7013 [0.6691, 0.7344] | ***0.7126 [0.6947, 0.7301]*** | 0.6742 [0.6417, 0.7057] | ***0.7162 [0.6995, 0.7327]*** | 0.7365 [0.7151, 0.7588] |
| **PowCou** | 1s | 0.8048 [0.7696, 0.8384] | 0.7873 [0.7529, 0.8215] | 0.5351 [0.5232, 0.5480] | 0.7508 [0.7042, 0.7952] | 0.6178 [0.5948, 0.6434] | 0.6000 [0.5756, 0.6268] |
|  | 2.5s | 0.8751 [0.8597, 0.8898] | 0.8667 [0.8532, 0.8797] | 0.5814 [0.5670, 0.5958] | 0.8467 [0.8294, 0.8630] | 0.6694 [0.6557, 0.6842] | 0.6798 [0.6651, 0.6953] |
|  | 5s | 0.9056 [0.8913, 0.9192] | 0.8906 [0.8761, 0.9048] | 0.6113 [0.5952, 0.6266] | 0.8808 [0.8640, 0.8968] | 0.7146 [0.6994, 0.7299] | 0.7261 [0.7109, 0.7413] |
|  | **10s** | 0.9291 [0.9163, 0.9415] | 0.9227 [0.9097, 0.9348] | ***0.6637 [0.6493, 0.6780]*** | 0.8941 [0.8738, 0.9132] | ***0.7354 [0.7201, 0.7503]*** | 0.7594 [0.7458, 0.7726] |

**A4 Robustness Analysis: dwPLI-based Classification**

**A4.1 Methods**

To assess whether the advantage of phase-base connectivity measures is robust to volume conduction artifacts, we computed the debiased weighted Phase Lag Index (dwPLI) as an alternative phase-based connectivity measure for all electrode pair and frequency band combinations (Vinck et al., 2011). dwPLI quantifies the asymmetry of the cross-spectral phase-angle distribution, suppressing contributions from zero-lag and near-zero-lag coupling that may arise from common sources. It is defined as:

$$\begin{aligned} \mathrm{dwPLI}_{\mathrm{xy}}=\frac{\left[ \frac{1}{n}\Sigma\left( C_{xy}\left( t \right) \right)^{2}-\frac{1}{n}\Sigma\left| Im\left( C_{xy}\left( t \right) \right) \right| \right]}{\left( \frac{1}{n}\Sigma\left| Im\left( C_{xy}\left( t \right) \right) \right| \right)^{2}}\#\left( A6 \right) \end{aligned}$$

where $Im\left( C_{xy}\left( t \right) \right)$ denoted the imaginary part of the cross-spectrum between signals x and y at time point t, and n is the number of time points. The debiasing term removed the positive bias of the standard weighted PLI that arises with finite sample sizes.

dwPLI-based functional connectivity and CFC features were computed following the same band-pass filtering and Hilbert transform pipeline as the primary analysis, using 10-second trial segments and 100 NCA-selected features. Classification accuracy was compared against the corresponding 10-second trial accuracy of all other feature types using the method in Section 2.5.3. With the selected dwPLI-based features, we also applied the topographical analysis in Section 2.4.3 to evaluate if they share the spatial patterns revealed by ISPC or spectral coherence in Figure 6.

**A4.2 Results**

Table A3 shows that dwPLI-based functional connectivity and CFC both classified experimental conditions above chance (50.00%), with mean accuracies of 71.09% ± 14.44% and 60.85% ± 10.21% respectively. However, none of the measure was significantly better than ISPC-based or power-based features (Table A4), suggesting that dwPLI does not improve upon existing measures for pain discrimination, even combined with CSD preprocessing.

Despite the lower classification performance, the topographic analysis revealed that dwPLI-based functional connectivity identified frontal-occipital long-range alpha-phase connectivity as a pain-specific signature. Specifically, connectivity between FP2 and PO6/PO8 was consistently selected when discriminating the pain condition from all non-painful conditions (warm and resting states; Figure A2), a pattern broadly consistent with the frontal-occipital connectivity identified by ISPC in the main analysis (Figure 6). Although the specific electrode pairs differ from those identified by ISPC, the preservation of the frontal-occipital spatial structure under zero-lag suppression by dwPLI confirms that the long-range phase connectivity finding is not solely attributable to reference effects or residual volume conduction.

**Table A3.** **Mean classification accuracy ± their standard deviation of dwPLI-based features in %.**

| Feature Type | OvsC | OvsH | OvsW | CvsH | CvsW | HvsW |
| --- | --- | --- | --- | --- | --- | --- |
| dwPLI Con | 76.95 ± 14.18 | 63.68 ± 9.13 | 63.85 ± 8.93 | 82.21 ± 10.90 | 81.83 ± 9.49 | 58.07 ± 11.60 |
| dwPLI Cou | 60.51 ± 11.88 | 60.14 ± 7.08 | 59.87 ± 7.07 | 65.56 ± 12.05 | 65.24 ± 11.94 | 53.80 ± 3.06 |

**Table A4.** **Summary of post-hoc pairwise comparisons of prediction accuracy across feature types.** The post-hoc comparisons including non-dwPLI features are ignored in this table, which are equivalent with the results in Table 1. dwPLICon represents dwPLI-based functional connectivity at the alpha band, and dwPLICou represents the dwPLI-based cross-frequency coupling across delta, theta, alpha and low beta bands.

| **Feature 1** | **Feature 2** | **Pairwise comparison between factor levels** | | | | |
| --- | --- | --- | --- | --- | --- | --- |
|  |  | **Difference** | **SE** | **t** | **df** | **p** |
| PhaCou | dwPLICon | 0.1649 | 0.0295 | 5.58 | 25 | ***<.001 |
| PhaCou | dwPLICou | 0.2674 | 0.0295 | 9.05 | 25 | ***<.001 |
| PhaCon | dwPLICon | 0.1194 | 0.0295 | 4.04 | 25 | **<.01 |
| PhaCon | dwPLICou | 0.2219 | 0.0295 | 7.51 | 25 | ***<.001 |
| PowCou | dwPLICon | 0.1064 | 0.0295 | 3.60 | 25 | *<.05 |
| PowCou | dwPLICou | 0.2089 | 0.0295 | 7.07 | 25 | ***<.001 |
| PowCon | dwPLICon | -0.0054 | 0.0295 | -0.18 | 25 | 1 |
| PowCon | dwPLICou | 0.0970 | 0.0295 | 3.29 | 25 | *<.05 |
| dwPLICon | dwPLICou | 0.1024 | 0.0295 | 3.47 | 25 | *<.05 |


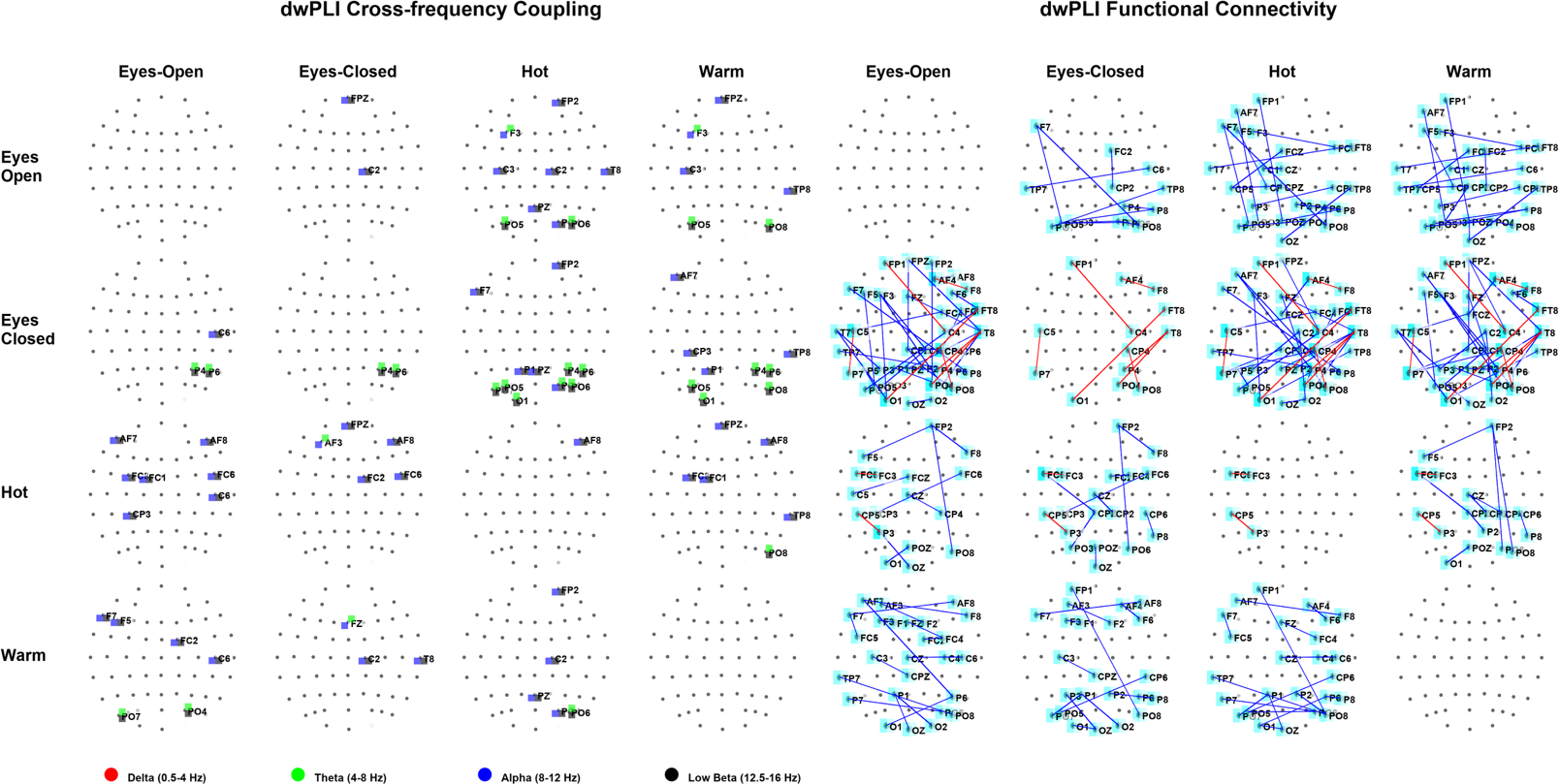


**Figure A2.** **Topographical distribution of dwPLI-based neural signatures**. Except for the diagonal from top-left to right-bottom, the row represents one condition, the column represents the other condition in the binary classification. (1) In the cross-frequency coupling patterns, the involved frequency bands are marked in different colours, which are presented in the legend. (2) In the functional connectivity, features that are shared by at least two classifiers were marked with blue lines. Features shared by all classifiers of a particular row were marked in red (only these features were marked on the diagonal).

**A5 Cross-Condition Evaluation of Binary Classification**

**A5.1 Methods**

To assess whether the classifiers capture neural signatures specific to pain or the differences between thermal sensations and cognitive components, we conducted a cross-condition evaluation. Models trained on one binary classification were tested on a related classification that shared one condition, and the other condition shared a characteristic (i.e., pain or thermal sensation). Using 10-second epochs and 100 selected features by NCA, we defined controlled transfer pairs as: (1) For pain detection: H vs. W is paired with H vs. O, since W and O are both non-painful conditions with eyes open; (2) for thermal pain detection: H vs. O is paired with H vs. C, since O and C both lack thermal stimulation; (3) For thermal sensation detection: H vs. O is paired with W vs. O, and H vs. C is paired with W vs. C, where H and W shared thermal stimulation while O and C shared its absence. Figure A3 displays an example of the transfer.

For each transfer pair, the SVM trained on one classification (as in Section 2.5.2) was applied to the other, and the resulting accuracy (i.e., transfer accuracy) was compared with the standard within-classification accuracy (i.e., standard accuracy) using paired t-tests across 36 participants. Bonferroni correction was applied to account for multiple comparisons within each feature type.


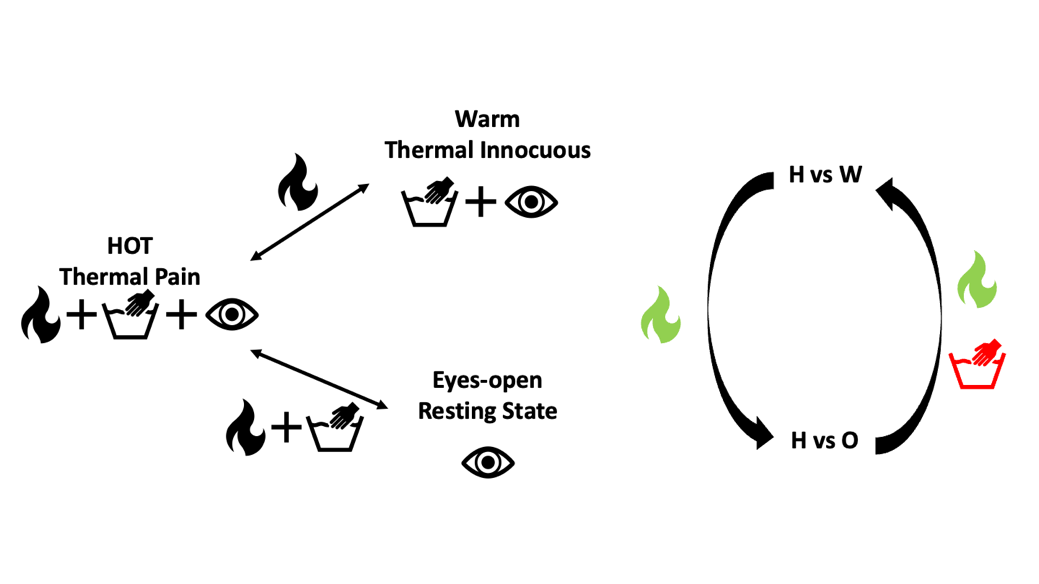


**Figure A3. An example schematic of the cross-condition transfer analysis.** Left: The HOT thermal pain condition (H) shares overlapping sensory components with two non-painful control conditions: Warm thermal innocuous (W), which shares both thermal stimulation and visual cognition, and Eyes-open resting state (O), which shares only visual input. Right: Transfer design between the two classification pairs. A classifier trained on H vs. W was tested on H vs. O, and vice versa. Transfer from H vs. W to H vs. O preserved classification performance (indicating that the H vs. W model captures pain-specific signatures), whereas transfer from H vs. O to H vs. W significantly degraded performance (indicating that the H vs. O model relies more heavily on thermal sensation features versus visual cognition).

**A5.2 Results**

Table A5 and Figure A4 present the transfer accuracy and statistical comparisons for all four feature types. For both power- and phase-based features (functional connectivity and CFC), transfer from H vs. W to H vs. O showed no significant accuracy loss (p >.05), whereas the reverse transfer, i.e., from H vs. O to H vs. W, significantly degraded performances (all **p<.01). This asymmetry indicates that the H vs. W classifier captured pain-specific signatures that generalise to other pain vs. non-pain contrasts. But H vs. O classification relied more heavily on thermal sensations’ signatures, which does not transfer when thermal stimulation is present in both conditions, i.e., H vs. W. For thermal sensation detection, transfer between H vs. O and W vs. O, or between H vs. C and W vs. C was largely non-significant for power-based features and phase-based functional connectivity (p>.05). It indicated that these classifiers captured shared thermal processing signatures that generalise across pain (H) and non-painful (W) states.

These findings support the interpretation that connectivity features extracted from H vs. W classification which was absent in the contrast between pain and resting states, such as the C1-AF7 alpha-phase connectivity, can reflect pain-specific neural integration rather than general thermal processing.


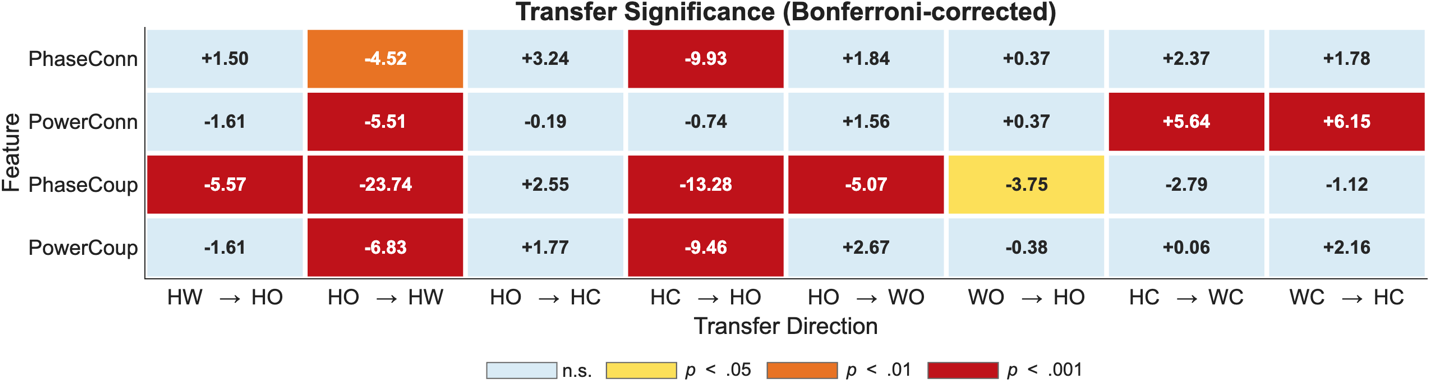


**Figure A4. Cross-condition transfer accuracy for binary classification.** Cell colour indicates the Bonferroni-corrected significance of paired t-tests comparing transfer accuracy to standard (within-condition) accuracy. t-values are displayed within each cell, where positive values indicate transfer accuracy exceeding standard accuracy, and negative values indicate the converse. Rows correspond to feature types and columns to transfer directions (training condition → testing condition).

**Table A5.** **Cross-condition transfer accuracy for four feature types.** Each row reports one transfer direction (e.g., HW_to_HO denotes a model trained on H vs. W and tested on H vs. O). Transfer accuracy (mean ± standard deviation) is compared against the standard within-classification accuracy of the training conditions, matched the same 36 participants, Bonferroni-corrected p-values are reported. (PhaseConn: Alpha-phase functional connectivity, PowerConn: Power-based function connectivity, PhaseCoup: Alpha-phase CFC, PowerCoup: Power-based CFC)

| **Feature** | **Transfer**  **(Train_to_Test)** | **N** | **Training acc % (mean±std)** | **Testing acc % (mean±std)** | **t** | **p-value**  **(Bonferroni)** |
| --- | --- | --- | --- | --- | --- | --- |
| PhaseConn | HW_to_HO | 36 | 68.98 ± 11.27 | 72.42 ± 11.11 | 1.50 | >.05 |
|  | HO_to_HW | 36 | 74.72 ± 10.36 | 62.76 ± 10.82 | -4.52 | **<.01 |
|  | HO_to_HC | 36 | 74.72 ± 10.36 | 83.37 ± 14.35 | 3.24 | >.05 |
|  | HC_to_HO | 36 | 93.30 ± 8.28 | 66.58 ± 9.67 | -9.93 | ***<.001 |
|  | HO_to_WO | 36 | 74.72 ± 10.36 | 79.01 ± 14.01 | 1.84 | >.05 |
|  | WO_to_HO | 36 | 77.00 ± 9.96 | 77.91 ± 13.90 | 0.37 | >.05 |
|  | HC_to_WC | 36 | 93.30 ± 8.28 | 96.09 ± 5.49 | 2.37 | >.05 |
|  | WC_to_HC | 36 | 93.04 ± 8.06 | 95.50 ± 6.91 | 1.78 | >.05 |
| PowerConn | HW_to_HO | 36 | 71.17 ± 13.60 | 66.82 ± 12.05 | -1.61 | >.05 |
|  | HO_to_HW | 36 | 73.30 ± 11.73 | 60.20 ± 11.00 | -5.51 | ***<.001 |
|  | HO_to_HC | 36 | 73.30 ± 11.73 | 72.70 ± 16.88 | -0.19 | >.05 |
|  | HC_to_HO | 36 | 66.81 ± 24.64 | 63.37 ± 9.14 | -0.74 | >.05 |
|  | HO_to_WO | 36 | 73.30 ± 11.73 | 77.86 ± 12.43 | 1.56 | >.05 |
|  | WO_to_HO | 36 | 73.35 ± 17.51 | 74.86 ± 13.04 | 0.37 | >.05 |
|  | HC_to_WC | 36 | 66.81 ± 24.64 | 91.17 ± 10.37 | 5.64 | ***<.001 |
|  | WC_to_HC | 36 | 68.86 ± 23.76 | 93.38 ± 7.21 | 6.15 | ***<.001 |
| PhaseCoup | HW_to_HO | 36 | 74.10 ± 10.77 | 60.48 ± 16.16 | -5.57 | ***<.001 |
|  | HO_to_HW | 36 | 83.49 ± 8.89 | 53.32 ± 4.71 | -23.74 | ***<.001 |
|  | HO_to_HC | 36 | 83.49 ± 8.89 | 89.23 ± 10.33 | 2.55 | >.05 |
|  | HC_to_HO | 36 | 96.86 ± 4.02 | 67.20 ± 10.56 | -13.28 | ***<.001 |
|  | HO_to_WO | 36 | 83.49 ± 8.89 | 76.90 ± 9.98 | -5.07 | ***<.001 |
|  | WO_to_HO | 36 | 84.89 ± 9.87 | 77.99 ± 11.53 | -3.75 | *<.05 |
|  | HC_to_WC | 36 | 96.86 ± 4.02 | 94.04 ± 8.45 | -2.79 | >.05 |
|  | WC_to_HC | 36 | 96.29 ± 4.77 | 95.66 ± 5.73 | -1.12 | >.05 |
| PowerCoup | HW_to_HO | 36 | 67.84 ± 10.04 | 63.50 ± 13.16 | -1.61 | >.05 |
|  | HO_to_HW | 36 | 73.87 ± 10.80 | 57.45 ± 10.42 | -6.83 | ***<.001 |
|  | HO_to_HC | 36 | 73.87 ± 10.80 | 80.03 ± 17.06 | 1.77 | >.05 |
|  | HC_to_HO | 36 | 93.58 ± 8.98 | 64.61 ± 11.05 | -9.46 | ***<.001 |
|  | HO_to_WO | 36 | 73.87 ± 10.80 | 79.96 ± 11.60 | 2.67 | >.05 |
|  | WO_to_HO | 36 | 77.62 ± 8.43 | 76.62 ± 14.14 | -0.38 | >.05 |
|  | HC_to_WC | 36 | 93.58 ± 8.98 | 93.66 ± 8.42 | 0.06 | >.05 |
|  | WC_to_HC | 36 | 92.61 ± 8.79 | 94.98 ± 6.25 | 2.16 | >.05 |

**(a)**


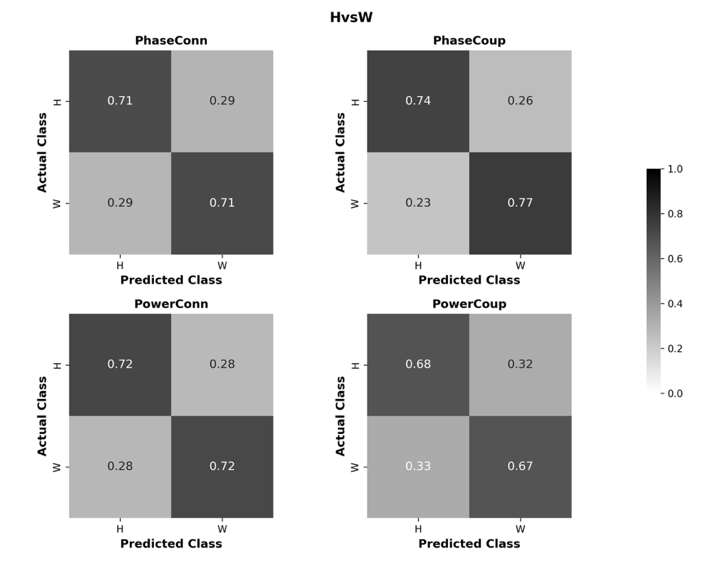


**(b)**


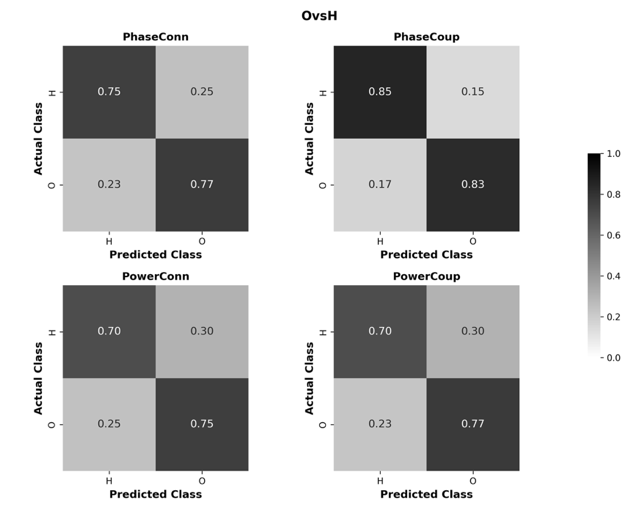


**(c)**


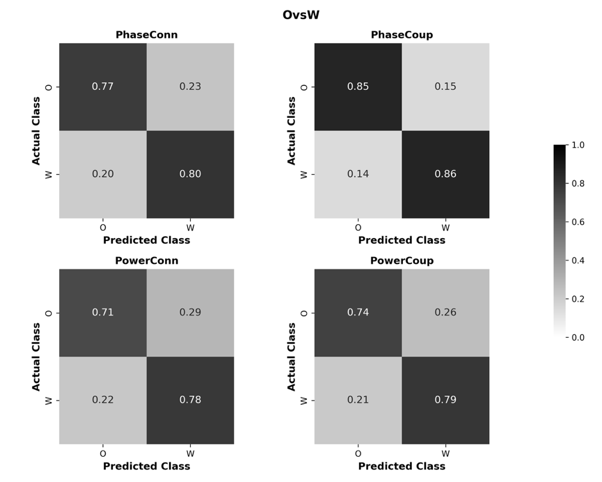


**Figure A5. Confusion matrices of binary classifications of interest from 10-second trials.** There are two types of classifications of interest, including pain (H) vs. non-painful (O/W) and resting state (O) vs. thermal stimuli (H/W). Each figure involves the confusion matrices produced by different neural signatures, where 'Conn' represents functional connectivity, and 'Coup' represents cross-frequency coupling (CFC). Totally, phase-based CFC was the best signature in all classifications of interest.


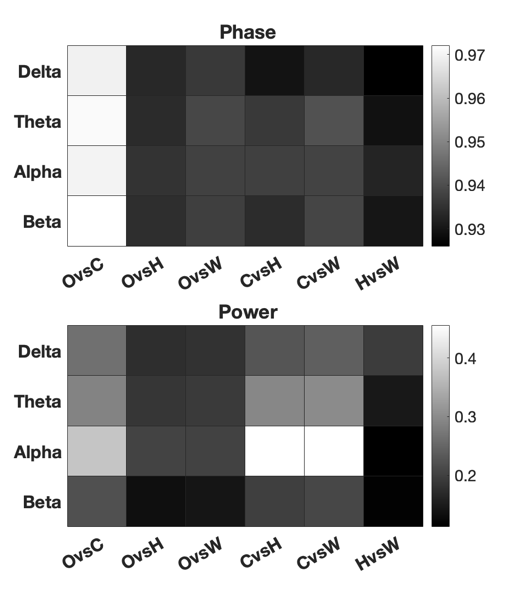


**Figure A6. Heatmaps illustrating the mean Kullback–Leibler divergence (KLD) for z-scored exponential phases (Phase) and magnitude (Power) distributions.** The KLDs were produced across four frequency bands (Delta, Theta, Alpha, Beta) and six condition comparisons (OvsC, OvsH, OvsW, CvsH, CvsW, HvsW). Each cell shows the mean KLD value across 62 channels $\times$ 36 participants for the corresponding frequency band and condition contrast. Darker shades denote higher divergence (i.e., greater discrepancy) between the compared distributions. For observation, the KLDs were rescaled between 0 and 1 according to the maximum and minimum KLDs from both measures.
